# Unilateral inferior oblique anterior transposition for markedly asymmetric dissociated vertical deviation with unilateral inferior oblique over-action

**DOI:** 10.1186/s12886-019-1205-z

**Published:** 2019-08-28

**Authors:** Shuang-Qing Wu, Qi-Bin Xu, Wen-Yan Sheng, Li-Wei Zhu

**Affiliations:** Department of Ophthalmology, Zhejiang Medicine and Western Medicine integrated Hospital (Hangzhou Red-Cross Hospital), 208 Huancheng Road East, Hangzhou, 310003 Zhejiang China

**Keywords:** Inferior oblique anterior transposition, Inferior oblique over-action, Dissociated vertical deviation, Unilateral surgery

## Abstract

**Background:**

To evaluate the results of unilateral inferior oblique anterior transposition (IOAT) for markedly asymmetric dissociated vertical deviation (DVD) combined with inferior oblique over-action (IOOA).

**Methods:**

Retrospective chart review of the records of all patients with asymmetric DVD combined with unilateral IOOA in the non-dominant eye who received unilateral IOAT on the non-dominant eye. No other muscles were operated on simultaneously. The amount of DVD and IOOA were measured before and after the operation and statistically analysed.

**Results:**

Seventeen patients were included. The mean age at surgery was 23.5 ± 8.4 (range 12–38) years old. The mean postoperative follow-up period was 15.7 ± 7.2 (range 6–32) months. The primary position DVD was 19.6 ± 5.4 (range 14–36) PD preoperatively and decreased significantly to 2.9 ± 2.0 (range 0–8) PD postoperatively (*P* < 0.01). Preoperatively, there were 2, 7, and 8 patients with + 1, + 2, and + 3 IOOA, respectively, and these were reduced from 2.4 ± 0.7 to 0.3 ± 0.4 postoperatively (*P* < 0.01). None of the patients were complicated obvious hypotropia, anti-elevation syndrome or IOOA in the contralateral eye.

**Conclusions:**

Unilateral IOAT was recommended in patients with asymmetric DVD coexists with unilateral IOOA.

## Background

Dissociated vertical deviation (DVD) is an ocular motor disorder characterized by upward drifting of one or both eye when the other eye is fixing on a target. It represents an abnormal vertical movement of eye ball that is contradictory to the role of nerve domination [1]. When DVD coexisted with inferior oblique over-action (IOOA), inferior oblique anterior transposition (IOAT) is recommended [2–6]. It was considered to be an ideal method to reduce the strength of the inferior oblique muscle and simultaneously restrict the floating phenomenon of DVD. Most clinicians suggested that binocular DVD should be treated with binocular surgery, especially for symmetric DVD [3, 5, 7]. However, treatment decisions are more complex in asymmetric DVD, in which bilateral asymmetric [8–10] or unilateral [4] surgery may be considered. Due to the possibility of adverse outcomes, such as hypotropia and contralateral DVD, bilateral surgery was more accepted by clinicians. Nevertheless, unilateral IOAT has also achieved appreciable outcomes in some studies [4, 6, 9]. We considered that this surgical design could achieve promising results while causing the least damage. The purpose of this study was to analyse the outcomes and complications observed in a cohort of patients who underwent unilateral IOAT for the treatment of markedly asymmetric DVD combined with unilateral IOOA.

## Methods

A retrospective chart review was conducted in all patients who received unilateral IOAT for DVD at Hangzhou Red-Cross Hospital between March 2015 and September 2017. Subjects with significant asymmetric DVD, manifest DVD of 10 PD or more in at least one eye, unilateral IOOA, and receiving surgery on the non-dominant eye with IOOA were included. A minimum postoperative follow-up period of 6 months was included in the analysis. Patients were excluded if they have no IOOA or alternate fixation. Patients with paralytic strabismus, previous strabismus surgery, neurologic, genetic, or craniofacial abnormalities were also excluded.

In addition to regular ophthalmological examinations, DVD was measured using the prism and alternate cover test with the eyes in the primary position, fixating on an accommodative target at a far (6-m) and near (1/3-m) distance to evaluate deviations in the primary position with full refractive correction. The fixating eye was changed to evaluate the difference in vertical deviation between the eyes. A difference of 5 PD or more was defined as asymmetric DVD. The degree of IOOA was measured in the field of action of inferior oblique and estimated from + 1 to + 4, as previously described [10].

All surgeries were performed by the same surgeon (LWZ). In all patients, the eye with more DVD underwent IOAT. The procedure was performed via a temporal inferior fornix approach with a 6 mm conjunctival incision. Tenon’s capsule was incised and the sclera was exposed. The lateral and inferior recti were isolated by muscle hooks to maintain the eye in a position of elevation and adduction. The distal end of the inferior oblique muscle was identified and imbricated with 6–0 Vicryl sutures and dissected from the sclera. The inferior oblique was transported and fixed on the same horizontal superficial sclera adjacent to the inferior rectus insertion, at a location 1 mm posterior to the inferior rectus insertion for + 1 to + 2 IOOA, at the level of the insertion for + 3 IOOA, and 1 mm anterior to the insertion for + 4 IOOA. The muscle was reattached to the sclera using a crossed sword technique. The overlying conjunctiva was closed with interrupted 8–0 Vicryl sutures. Horizontal extraocular muscle surgery to correct congenital esotropia or exotropia was performed with a secondary operation at 3 months after IOAT.

Postoperative follow-ups were performed after 1 week, 1 month, and 3 months, and the patient was then observed every 3–6 months, on average. The results of the examination performed at the last visit was used for analysis. A successful outcome was defined as a residual DVD of less than 10 PD in the primary position and an IOOA of ≤ + l.

Continuous variables were expressed as the mean and standard deviation and were compared using two-sided student’s *t*-test. Discrete various were compared using the Wilcoxon Signed Ranks test before and after the operation. A *P*-value < 0.05 was considered statistically significant.

## Results

Seventeen subjects met the study inclusion criteria and the mean age at surgery was 23.5 ± 8.4 years old (range, 12 to 38 years old). Among these individuals, there were 6 males and 11 females. Two patients had congenital exotropia, and 5 patients had esotropia. The mean postoperative follow-up period was 15.6 ± 7.2 months (range, 6 to 32 months). Preoperatively, the primary position of DVD was 19.6 ± 5.4 PD (range, 14 to 36 PD), and there were 2, 7, and 8 patients with + 1, + 2, and + 3 IOOA, respectively (Table 1).

**Table 1:** Pre- and postoperative information of DVD and IOOA in all reported cases

Patient No.	Age	BCVA	Pre-OP DVD (R/L, PD)	Pre-OP IOOA (R/L)	Post-OP DVD (R/L, PD)	Post-OP IOOA (R/L)	Follow-up(mos)
1	12	0.6/1.0	15/4	2/0	0/4	0/0	9
2	14	0.6/0.8	18/6	1/0	2/7	0/0	27
3	25	0.4/0.8	23/4	3/0	2/4	0.5/0	8
4	18	1.0/0.6	2/14	0/2	2/0	0/0	19
5	16	0.8/0.5	6/17	0/2	6/4	0/0	18
6	32	1.0/0.8	4/16	0/2	4/2	0/0	18
7	38	0.3/0.8	25/8	3/0	2/8	1/0	16
8	34	0.8/0.4	4/36	0/3	4/5	0/1	15
9	13	0.6/0.8	18/6	1/0	2/6	0	14
10	17	0.6/1.0	16/4	2/0	2/4	0	16
11	33	0.3/0.8	23/8	3/0	8/8	1/0	11
12	15	0.8/0.8	6/15	0/2	6/4	0/0	10
13	27	1.0/0.8	4/17	0/3	6/2	0/0.5	26
14	22	0.4/1.0	24/8	3/0	6/8	0/0	9
15	29	0.8/1.0	16/6	2/0	2/6	0/0	32
16	31	0.8/0.5	8/21	0/3	8/4	0/0.5	6
17	23	0.8/0.5	6/19	0/3	6/4	0/0	11

*BCVA* best corrected visual acuity, *DVD* dissociated vertical deviation, *PD* prism degree, *IOOA* inferior oblique over-action

Postoperatively, the primary position of DVD was reduced to 2.9 ± 2.0 PD (range 0 to 8 PD), which was significantly different from preoperative DVD (*P* < 0.01). Compared with the preoperative mean value of 2.4 ± 0.7, postoperative IOOA decreased significantly to 0.3 ± 0.4.

**Fig. 1:**
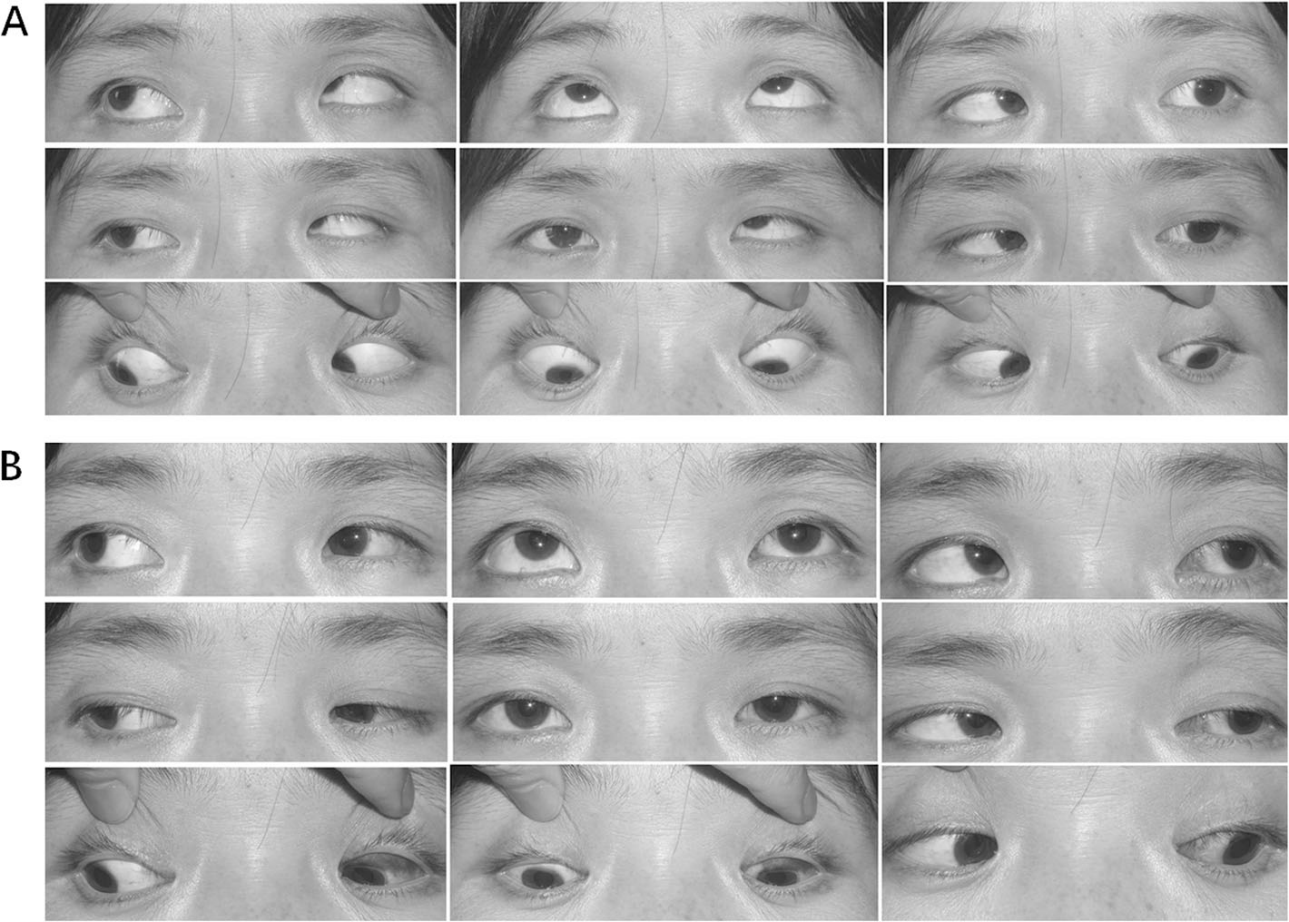
A representative patient with DVD coexisted with IOOA who underwent unilateral inferior oblique anterior transposition in the left eye. **a**. Preoperative findings in the patient. Note + 3 IOOA and 36 PD DVD in the left eye. **b**. Postoperative findings show + 1 IOOA and 5 PD DVD in the left eye. (Note: Approval consent for publication of the photo was attained from the patient herself)

Postoperatively, none of the patients developed obvious hypotropia, anti-elevation syndrome or IOOA in the contralateral eye postoperatively, and no changes were noted in contralateral eye DVD, IOOA, diplopia, and fixing properties. DVD and IOOA were successfully corrected in all cases, and a characteristic patient example was shown in Fig. 1.

## Discussion

DVD is a dissociated strabismus characterized by upward drifting of one or both eyes when binocularity is interrupted. DVD is usually bilaterally. Asymmetric DVD is probably associated with unequal IOOA or superior rectus overaction [7]. The management of DVD is challenging for strabismus surgeons. Multiple approaches have been proposed for the treatment of DVD, but there is no agreement among surgeons regarding the best practice [7]. However, when DVD coexisted with IOOA, IOAT is preferred by most clinicians as it reduces the IOOA while simultaneously restricting the superior floating phenomenon of DVD [2–4, 8, 11, 12]. Full IOAT includes the posterior fibers with J deformity that form a neurofibrovascular bundle. The neurovascular bundle provides the inferior oblique muscle with a new functional origin and convert the inferior oblique muscle from an elevator to a depressor. The depressor effect is likely due to a combination of active contraction of the distal inferior oblique muscle and mechanical restriction on the elevation of the eye [10, 13].

A number of reports have showed satisfactory clinical results for the IOAT procedure in DVD with IOOA [2–4, 11]. Symmetric DVD was always treated with symmetric surgery, whereas asymmetric DVD or IOOA was preferred for bilateral asymmetric surgery or unilateral surgery. Snir et al. [10] suggested the use of bilateral IOAT with monocular-graded inferior oblique resection for asymmetric DVD with IOOA as this procedure could improve DVD more than that can be achieved by equal bilateral IOAT. Pineles et al. [8] used bilateral asymmetric IOAT to treat incomitant asymmetric DVD and showed that this procedure led to improvements in incomitant DVD, a V-pattern and IOOA. Rajavi et al. [11] demonstrated both graded and ungraded asymmetric binocular IOAT could effectively reduce DVD and IOOA. Nevertheless, a unilateral surgical procedure was used only in patients with unilateral markedly asymmetric DVD. Burke [6] observed that unilateral IOAT achieved satisfactory results for DVD with IOOA when the preoperative DVD was less than 15 PD. However, Bothun and Summers [4] considered unilateral IOAT could be an effective treatment for unilateral or markedly asymmetric DVD in patients with a strong, contralateral fixation preference. In their study, 9 patients with DVD measuring 17 to 33 PD were also successfully corrected. In our study, the patient’s inclusion criteria were similar to those described in Bothun’s study, in which all cases in a case series of patients with primary DVD measuring 14 to 36 PD in the operated eye were successfully treated for motility disturbance.

In the literature, when performing inferior oblique anterior transposition, the optimal placement of the muscle is various and controversial. The standard placement is at the temporal border of the inferior rectus muscle insertion. Kratz [14] performed graded procedures at a position 1 mm posterior to and 1 mm anterior to the temporal position of the inferior rectus insertion site depending on the severity of DVD. Seawright and Gole [15] performed graded IOAT to positions located 2 mm posterior and 2 mm anterior to the temporal position of the inferior rectus insertion site depending on the presence or amount of preoperative IOOA, V pattern, hypertropia, and DVD. Placing the inferior oblique at a position 2 to 4 mm anterior to the lateral end of the inferior rectus muscle insertion site did not increase the effectiveness of IOAT, but it might increase the risk of postoperative anti-elevation syndrome [16, 17]. Recently, Ford [3] and Farid [2] reported that anterior and nasal transposition of the inferior oblique might mechanically restrict the elevation of the eye and improve DVD more effectively than is achieved by IOAT; however, it may also induce hypotropia and consecutive horizontal strabismus. We performed the procedure at a placement position similar to that used by Kratz, but we graded for the position based on the degree of IOOA. In the monocular procedure, IOOA and DVD were significantly improved, and no cases developed obvious anti-elevation syndrome.

With the generalization IOAT, some side effects, such as hypotropia [6, 9] and anti-elevation syndrome have been mentioned [16, 17]. Although hypotropia and anti-elevation were also reported in unilateral IOAT surgery, its complications were often mild [4, 6]. In our study, IOOA and DVD were significantly reduced in all patients, and there were no related complications. This result is probably related to our inclusion criteria, which required unilateral IOOA, significantly different primary positions of DVD in both eyes, and surgery on the non-fixing eye. Because the non-fixing eye always occupied a higher position, performing IOAT on the non-fixing eye could improve the floating phenomenon observed in DVD with fewer complications. Moreover, the patients in our study had worse vision or amblyopia on the operated eye. Patients with paralytic strabismus or alternate fixation were excluded. These inclusion criteria may contribute to less postoperative impaction on the contralateral eye. Meanwhile, individual difference in the strength of the muscle may impact the efficiency of IOOA.

There are some limitations to this study. It is a retrospective study performed without a control group. The sample size was relatively small due to the specific inclusion and exclusion criteria applied in this study. The follow-up duration was not long enough to observe some delayed complications. Moreover, we did not evaluate the development of the V pattern or limitations on elevation during abduction. Concurrent horizontal muscle surgery was preferred in most of previous studies. However, we performed secondary horizontal muscle surgery at 3 months after IOOA, considering the impact of DVD and IOOA on horizontal strabismus. These factors make our results less comparable to those achieved in previous studies.

## Conclusions

According to this retrospective study, unilateral IOAT can be recommended in patients with markedly asymmetric DVD coexist with unilateral IOOA. Surgery should be performed on the non-dominant eye with IOOA.

## Data Availability

The datasets during the current study are available from the corresponding author on reasonable request.

## Abbreviations

DVD: Dissociated vertical deviation
IOAT: Inferior oblique anterior transposition
IOOA: Combined with inferior oblique over-action
PD: Prism degree
